# Melatonin Scavenger Properties against Oxidative and Nitrosative Stress: Impact on Gamete Handling and In Vitro Embryo Production in Humans and Other Mammals

**DOI:** 10.3390/ijms18061119

**Published:** 2017-06-14

**Authors:** Pía Loren, Raúl Sánchez, María-Elena Arias, Ricardo Felmer, Jennie Risopatrón, Carolina Cheuquemán

**Affiliations:** 1 Centro de Biotecnología de la Reproducción (BIOREN-CEBIOR), Facultad de Medicina, Universidad de La Frontera, Temuco 4811230, Chile; lorenreyesfran@gmail.com (P.L.); raul.sanchez@ufrontera.cl (R.S.); mariascea@gmail.com (M.-E.A.); ricardo.felmer@ufrontera.cl (R.F.); jennie.risopatron@ufrontera.cl (J.R.); 2 Departamento de Ciencias Preclínicas, Facultad de Medicina, Universidad de La Frontera, Temuco 4811230, Chile; 3 Departamento de Producción Agropecuaria, Facultad de Ciencias Agropecuarias y Forestales, Universidad de La Frontera, Temuco 4811230, Chile; 4 Departamento de Ciencias Agronómicas y Recursos Naturales, Facultad de Ciencias Agropecuarias y Forestales, Universidad de La Frontera, Temuco 4811230, Chile; 5 Departamento de Ciencias Básicas, Universidad de La Frontera, Temuco 4811230, Chile

**Keywords:** melatonin, antioxidants, RNS, ROS, embryo development, DNA integrity, DNA oxidation, gene expression

## Abstract

Oxidative and nitrosative stress are common problems when handling gametes in vitro. In vitro development in mammalian embryos is highly affected by culture conditions, especially by reactive oxygen species (ROS) and reactive nitrogen species (RNS), because their absence or overproduction causes embryo arrest and changes in gene expression. Melatonin in gamete co-incubation during in vitro fertilization (IVF) has deleterious or positive effects, depending on the concentration used in the culture medium, demonstrating the delicate balance between antioxidant and pro-oxidant activity. Further research is needed to better understand the possible impact of melatonin on the different IVP steps in humans and other mammals, especially in seasonal breeds where this neuro-hormone system highly regulates its reproduction physiology.

## 1. Introduction

### 1.1. Free Radicals on Reproduction (ROS/RNS)

The protocols for in vitro maturation, fertilization, and embryo culture in assisted reproductive techniques (ART) have been greatly improved during the last decade. However, only a few embryos produced by ARTs are capable of carrying out development to full term. This is mainly due to the lack of optimal in vitro conditions that cannot mimic the in vivo conditions, leading to several differences between both conditions such as increased levels of ROS or RNS [1]. Both free radicals are generated as sub-products in physiological processes where the oxygen consumption is produced in the electron transport chain during cellular respiration in the mitochondria [2]. 

There is a duality in the role of ROS and RNS. Physiological levels are needed in several contexts: ROS are necessary in human follicles to establish pregnancy [3], as potential markers in patients for predicting the success of in vitro fertilization (IVF) [4], during the in vitro maturation of oocytes [5], in the resumption of meiosis from diplotene arrested oocytes [6], for stimulating the release of intracellular Ca^+2^ in oocytes [7], and for stimulating mitogen-activated protein kinases (MAPKs) [8]. In sperm physiology, ROS participate in hyperactivation [9], sperm capacitation [10,11,12,13,14], tyrosine phosphorylation [15], and the acrosome reaction [16].

RNS, on the other side, are necessary for the development of large antral follicles [17,18], to stimulate meiotic maturation in oocytes [19,20], in the ovulatory process [21], in early folliculogenesis up to the maturation step [22] and in preimplantation embryonic development [23,24]. Additionally, RNS participate in sperm capacitation [25,26,27] and the acrosome reaction [28]. 

When an imbalance between pro-oxidant molecules occurs due to the increase of ROS/RNS levels or the reduction of the antioxidant defense mechanisms, the phenomenon called oxidative or nitrosative stress is triggered [29,30].

### 1.2. Oxidative Stress

Oxygen (O_2_) is an essential element for aerobic organisms because oxidative metabolism represents the main energy source. The partial reduction of O_2_ results in ROS formation; these are molecules that contain one oxygen atom in their structure and possess at least one highly reactive unpaired electron in an outer orbital [31]. These molecules include two major groups: free radicals such as the superoxide anion (^•^O_2_^−^) and hydroxyl radical (^•^OH), and molecules such as hydrogen peroxide (H_2_O_2_) [32]. The production of ^•^O_2_^−^ is the initial step for the formation of ROS, which is generated by the acceptance of an electron by O_2_, catalyzed by NADPH oxidase or xanthine oxidase. This radical can be converted into H_2_O_2_ by the action of the superoxide dismutase (SOD) enzyme, and then degraded to H_2_O and O_2_ by catalase or glutathione peroxidase [33]. ^•^OH is generated during the Haber-Weiss reaction which produces more toxic free radicals through the interaction between ^•^O_2_^−^ and H_2_O_2_ [29]. Alternatively, two reactions using iron ions (Fe^+3^ and Fe^+2^), the Fenton reaction, can also generate ^•^OH [29].

In pathological events, ROS have been involved in patients with endometriosis [34]. The total amount of ROS in culture medium is negatively related with embryo implantation potential [35] or pregnancy [36]. High levels are correlated with poor oocyte quality [37] and cell meiotic arrest [6]. Previously, we investigated the induction of stress tolerance in bovine cumulus oocyte complexes (COCs) to generate oxidative stress resistance by incubation with H_2_O_2_ during in vitro embryo production [38]. We observed that exposing COCs to low H_2_O_2_ levels could induce stress tolerance in these embryos, determined by the embryo development, quality, and gene expression pattern [39].

### 1.3. Nitrosative Stress

Like ROS, RNS such as nitric oxide (NO) act as signaling molecules modulating various aspects of the reproductive physiology [40]; they influence and mediate the gametes and crucial reproductive processes such as sperm–oocyte interaction, implantation, and early embryo development [41]. Nitric oxide (NO) is generated either by enzymes, including neuronal nitric oxide synthase (nNOS), endothelial nitric oxide synthase (eNOS), and inducible nitric oxide synthase (iNOS) [40,42], or by a non-enzymatic pathway from nitrite involving hydrogen peroxide and d-or l-arginine [40]. High and sustained levels of RNS result in nitrosative stress with negative consequences for cells [40], leading to different pathologies [43]. The chemical reactivity of NO is rather low, but it reacts with ^•^O_2_^−^ yielding peroxynitrite (ONOO^−^), which is a potent oxidant inducing protein, lipid, and DNA damage [44].

Previously, we investigated nitrosative stress tolerance in oocytes by the in vitro incubation of oocytes adding NO donors during in vitro embryo production in bovine. However, no differences in the embryo quality or resistance to nitrosative stress were observed for incubation with either added 3-morpholinosidnonimine (SIN-1) [45] or added sodium nitroprusside (SNP) [46].

Currently, the administration of antioxidants is recommended for counteracting oxidative and nitrosative stress in cells. This review synthesises the experimental data that has been published and the advances in the knowledge on the effects of melatonin on gametes and the different steps of in vitro reproduction. Here, we argue that the application of melatonin should be considered for improving the efficiency and outcomes of reproductive biotechnologies in humans and domestic animals due to its capacity as a powerful antioxidant counteracting ROS/NOS induced damage.

### 1.4. Melatonin and ROS/RNS

Melatonin (*N*-acetyl-5-methoxytryptamine) is a multifunctional molecule secreted by the pineal gland in response to changes in light levels and other tissues [43,47]. Melatonin is produced at higher amounts by different tissues including Leydig cells, spermatocytes, and spermatids in testes [48]; in extrapineal organs, tissues, and fluids of mammals and humans [49,50]; and mast cells [51]. However, only melatonin secreted in the pineal gland plays an important role in the circadian sleep regulation [52] and reproductive function in seasonally breeding animals [53,54,55]. In humans, melatonin administration to IVF patients with sleep disorders improves the oocyte and embryo quality [56]. Additionally, the melatonin pattern secretion influences endocrine effects of the photoperiod, resulting in physiological alterations in reproduction [57]. It regulates the complex embryo-fetal developmental processes [58]. For example, the cold and dark winter periods in Norway may suppress the ovarian activity and estrus expression in cows, showing a higher reproductive performance during the summer months compared to the winter season [59], agreeing with the low in vitro embryo production rates we observed during the winter season in our geographic zone [60]. Circadian genes such as CLOCK, BMAL1, CRY1, CRY2, PER1, and PER2 are expressed and function as maternal mRNA regulating events in the oocytes and preimplantation embryos [61], and are involved in physiological processes, such as meiosis [62].

Melatonin is a potent free radical scavenger [52,63], directly reducing the ROS concentration and preventing the depletion of endogenous antioxidant enzymes [64]. The scavenging potential stems from it´s antioxidant reactions against ROS, including singlet oxygen, nitric oxide, hydrogen peroxide, and hydroxyl radical [65], and against RNS including nitric acid, peroxynitrite, and peroxynitrous acid [66]. Melatonin up-regulates the gene expression and activity of several antioxidant proteins [64,67,68,69], preserves optimal the mitochondrial function, and contributes to maintaining homeostasis against oxidative stress [70]. Its metabolites exhibit a powerful antioxidant capacity [44,71,72,73,74]. Melatonin readily combines with a superoxide releasing NO, thus preventing the formation of peroxynitrite, a free radical even more harmful than NO [75]. It has been described as a direct peroxynitrite scavenger [76].

**Figure 1:**
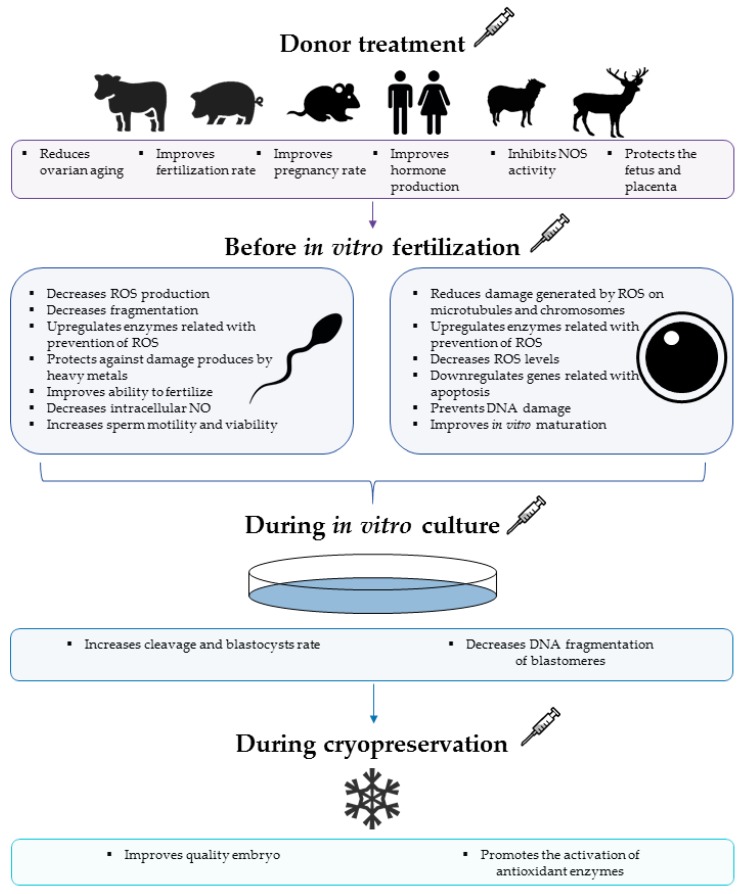
Effects of melatonin application in different steps on gametes and in vitro embryo production.

The effects of melatonin on gametes and in vitro production (Figure 1) for humans are summarized in Table 1 and for other mammals in Table 2, for data published between 2012 and 2016.

## 2. Melatonin Modulates Oxidative Stress on Gametes and In Vitro Embryo Production (IVP)

Its amphiphilic nature allows melatonin to pass biological barriers. This feature makes it an effective antioxidant, resulting in protections embryos of macromolecules against ROS [74,120]. In mammalian oocytes, melatonin can prevent damage generated by hypochlorous acid (HOCl) on spindle microtubules and chromosome alterations in metaphase-II mouse oocytes [121], upregulate *MnSOD* [90,122] and *Cu*-*ZnSOD* transcripts in cumulus cells [90], decrease ROS levels in oocytes [90], suppress Bax protein expression and decrease the Bax/Bcl-2 ratio in the ovaries [122], and prevent DNA damage [123] and nuclear fragmentation in cumulus cells [90]. Long term treatments with melatonin in humans have shown reductions of ovarian ageing, and increases in the litter size, pools of follicles, and in the telomere length [122]. Melatonin has also been shown to protect oocytes against the inhibitory effect of oxidative stress generated by H_2_O_2_ [123]. The protection results in an increased in vitro maturation rate [124], reduced oxidative damage in oocytes during in vitro maturation, and decreased mitochondrial activity [124]. The optimal mitochondrial membrane potential can be maintained by activating uncoupling proteins or by inhibiting the mitochondrial permeability transition pore [125]. The mechanism by which melatonin promotes oocyte maturation is not yet clear, but it is believed to be mediated via melatonin membrane receptors such as the melatonin receptor agonist IIK7 [109].

In human, intrafollicular concentrations of 8-OHdG and hexanoyl-lysine were significantly reduced by melatonin (3 mg/day) and vitamin E (600 mg/day) treatments [123]. The fertilization rate was improved by melatonin treatment compared to the previous IVF-ET cycle [123] and melatonin levels are associated with oocyte quantity and quality [122,126]. Melatonin improves progesterone production by corpus luteum in infertile women with a luteal phase defect [127].

Spermatozoa are sensitive to oxidative stress, leading to an apoptosis-like process. Melatonin can decrease mitochondrial ROS production when sperm is exposed to oxidative stress [128]. Thus, it is a powerful antioxidant and anti-apoptotic agent in ejaculated human spermatozoa by the inhibition of caspase-3 and caspase-9 activities [80,128,129]. Melatonin can prevent mitochondrial ROS formation under basal conditions and at an early time point upon oxidative stress induced by H_2_O_2_ exposure [130], increasing *MnSOD* expression [85], glutathione peroxidase [131], and glutathione reductase [132]; preventing DNA fragmentation [129], and therefore, improving sperm quality [130]. Additionally, the melatonin supplementation of semen extenders increases sperm motility and viability, and decreases ROS levels and lipid peroxidation [80], which improves the sperm quality after the freezing-thawing processes [133]. This antioxidant can protect from testicular injury induced by oxidative stress after cadmium (Cd) exposure [96]. Melatonin helps to protect sperm from ROS induced by cell sorting, a widely used technique for in vitro fertilization or artificial insemination [133].

Our experience during the supplementation of IVF medium with melatonin shows that this antioxidant has a dual effect over sperm function and embryo development in bovine [105]: lower concentrations (10–1000 nM) modulate the sperm quality by inducing changes in the sperm motility, increasing the Wobbler coefficient. On the other hand, a high melatonin concentration during sperm incubation (1000 nM) decreased the number of viable sperm with an intact acrosome membrane and induced high levels of DNA fragmentation and DNA oxidation. Similarly, high melatonin concentrations in IVF (0.01–1 mM) generated decreases in the blastocyst production rate, without affecting the embryo quality. During embryo culture, cells are exposed to higher oxygen concentrations, resulting in increased ROS production. Melatonin supplementation has a beneficial effect on in vitro fertilization in human patients [82,123] by improving the blastocyst formation rate and decreasing the DNA fragmentation of blastomeres [104].

Cryopreservation is a highly stressful process that significantly reduces the potential for embryo development. Melatonin added to the culture medium increases cleavage, blastocyst, and hatching rates [100,134]. It increases the total number of cells (TCN) [113] and trophectoderm (TE) cells, and the inner cell mass (ICM) ratio in vitrified embryos [134]. Melatonin reduces the apoptotic index [113,134], promotes the activation of antioxidant enzymes such as GST and SOD [100], decreases the level of oxidative substrates [100], and ameliorates the down-regulation of genes including NANOG and POU5F1, which are important for early embryo development [100].

## 3. Potential Use of Melatonin against Nitrosative Stress during ART

Melatonin has been described to reduce nitrosative/oxidative stress in many different tissues and organelles [74]. These data corroborate its protective effect against drugs, toxins, metals, and herbicides [135]. Melatonin acts on the NO/NOS system by reducing peroxynitrite formation in the brain during the first steps of the ischemic cascade, influencing the NO/NOS pathway and reducing oxidative and nitrosative stress [42]. High levels of NO are produced during acute renal failure by iNOS due to ROS/RNS activation, but can be counteracted by melatonin, attenuating lipid peroxidation and protein oxidation in the kidneys [136]. Similarly, melatonin administration counteracted iNOS activation and mitochondrial damage in the liver during sepsis [137]. Melatonin preserves fetal growth in rats through protection against ischemia/reperfusion-induced oxidative/nitrosative stress by preventing the oxidative damage in placental DNA and mitochondria [70]. Is has a neuroprotective due to counteracting i-mtNOS induction, oxidative stress, and mitochondrial dysfunction [138].

Melatonin has been shown to be protective for gamete handling in vitro. It has been proposed that melatonin inhibits the activity of the pro-oxidative enzyme nitric oxide synthase (NOS) in the Graafian follicle [55]. Melatonin delays ovarian ageing by multiple mechanisms, including an antioxidant action and by reducing the decline in oocyte quantity and quality in mice [139]. Therefore, melatonin could be useful against nitrosative stress due to the in vitro maturation of the oocytes.

A beneficial effect on male fertility has been described for humans and domestic animals: melatonin induces a significant decrease in the intracellular NO concentration in human sperm, increasing sperm motility and viability [43]. The NO concentration changes during the annual reproductive cycle in male adult buffalo, whereby NO is mainly present in the caput epididymis during short photoperiods coinciding with maximum gonadal activity [140]. According to this, and considering the influence of melatonin on the seasonal reproduction in these animals, we can suggest the potential use of melatonin to modulate NO levels to increase buffalo fertility or in other seasonal breeds, both during semen storage or IVF.

ARTs can induce vascular dysfunction and arterial hypertension related to epigenetic alterations of the regulation of the eNOS gene. However, this can be prevented by the addition of melatonin during the in vitro culture of embryos, which has doubled the success rate of IVF [141]. 

Melatonin has been found to protect the fetus and placenta from oxidative stress due to ROS and RNS [55]. Clinical melatonin treatment could be useful to increase or maintain umbilical blood flow by NO-dependent mechanisms in complicated pregnancies [75], as after embryo transfer of in vitro produced embryos (ET/IVP) in domestic animals.

Despite the fact that more specific studies regarding the melatonin effect against nitrosative stress in reproductive biotechnologies are scarce, melatonin has demonstrated direct and indirect beneficial effects against ROS. Therefore, considering that ROS can generate RNS, we can deduct that melatonin could have a protective action over nitrosative stress during gamete and embryo handling in the laboratory, as has been demonstrated in other tissues.

## 4. Conclusions

There is a long list of studies that support the use of melatonin against oxidative stress; however, much remains to be investigated regarding the role that melatonin might have on nitrosative stress during the in vitro manipulation and cryopreservation of gametes and embryos. Therefore, the evidence is clear that melatonin is involved in the protection against oxidative/nitrosative stress by scavenging free radicals, inducing the activity of antioxidant enzymes and preventing the induction of the mitochondrial pathway of apoptosis, improving gamete and embryo quality, both in human and domestic animals during ARTs.

## Figures and Tables

**Table 1:** Effect of melatonin on different steps of assisted reproductive techniques in humans.

Specie	Tissue	Treatment	Results	Reference
Human	Patient	3 mg per day from the fifth day onwards of one cycle in women with diminished ovarian reserve	Increases the mean number of M-II oocytes, top-quality embryos with grade 1 and 2	[77]
Human	Blastocyst	10^−7^ M in culture system in 3D (Encapsulation)	Increases the survival time of encapsulated embryos	[78]
Human	Patient	3 mg for 14 days in patients with polycystic ovarian syndrome	Enhances the oocyte and embryo quality	[79]
Human	Sperm	0.01 mM in freezing extender before cryopreservation of sperm from infertile men	Increases motility and viability, decreases ROS and MDA levels	[80]
Human	Patient	6 mg for 45 days	Increases the antioxidant capacity in seminal plasma, reduces the oxidative damage caused in sperm DNA, increases the quality of embryos	[81]
Human	Patient	3 mg/day for 2 weeks	Increases the fertilization rate I the second cycle, improves the fertilization and embryos quality rate	[82]

**Table 2:** Effect of melatonin in different steps of assisted reproductive techniques in other mammals.

Specie	Tissue	Treatment	Results	Reference
Porcine	Oocytes	10^−9^ M during in vitro maturation	Increases cleavage and blastocyst rate and the total cell number of blastocyst; promotes lipid metabolis, providing energy for oocyte maturation and embryo development	[83]
Rat	Animal	Intraperitoneal injection of 20 mg/kg for 4 weeks	Increases testosterone hormone in blood serum and body weigh	[84]
Mouse	Spermatogonial stem cells	10 mg/kg for 2 weeks after busulfan treatment	Relieves the loss and apoptosis in mouse testes; upregulates *MnSOD*	[85]
Mouse	Oocytes	10^−9^ to 10^−3^ M after in vitro maturation	Increases in vitro fertilization rate, reduces ROS and inhibits apoptosis	[86]
Bovine	Zygotes	1 µM for 3 h after insemination and at 40 °C	Reduces ROS levels in embryos	[87]
Mouse	Oocyte M-II	10^−9^ mol/L during vitrification/warming and PA	Increases blastocyst rate after warming compared with control group	[88]
Bovine	Sperm	10^−3^ M for 3 h before in vitro fertilization	Improves plasma membrane and acrosome integrity, mitochondrial activity; decreases intracellular ROS levels; increases the blastocyst rate and it decreases apoptosis rate	[89]
Bovine	Oocytes	10^−6^ or 10^−9^ M for 24 h during in vitro maturation	Up-regulates *MnSOD* and *Cu*-*ZnSOD* in cumulus cells; decreases fragmentation. Decreases ROS levels In oocytes	[90]
Bovine	Embryos	10^−7^ M melatonin for 24 h prior to exposure to 250 µM Paraquat (herbicide)	Decreases the incidence of apoptotic nuclei induced by Paraquat	[91]
Porcine	Oocytes	0.1 μM for 22–44 h after endoplasmic reticulum stress during in vitro maturation	Improves oocyte maturation and cumulus cells expansion induced by endoplasmic reticulum stress	[92]
Bovine	Oocytes	Melatonin-loaded lipid-core nanocapsules at 10^−6^ M, 10^−9^ M and 10^−12^ M during in vitro maturation	Enhances in vitro embryo production, decreases ROS levels and the apoptotic nuclei, upregulates *GPX1* and *SOD2* and downregulates *CASP3* and *BAX*	[93]
Bovine	Zygotes	Melatonin-loaded lipid-core nanocapsules at 10^−9^ M during in vitro culture	Increases hatching rate and embryo cell number, decreases cell apoptosis and ROS levels; downregulates *BAX*, *CASP3*, and *SHC1* genes, and upregulates *CAT* and *SOD2* genes	[94]
Mouse	Oocytes	10^−7^ M during in vitro maturation	Improves blastocyst rate and cell number of blastocysts	[95]
Mouse	Sperm	10 mg/kg body weight for 7 days during cadmium exposure	Reduces oxidative stress and inflammation induced by cadmium in male reproductive system	[96]
Mouse	Sperm	0.125 mg/mL in freezing extender during cryopreservation	Increases progressive motility, decreases ROS levels and upregulates *BCL-XL*	[97]
Buffalo	Oocytes	250 µM during in vitro maturation	Improves fertilization rate	[98]
Bovine	Oocytes	1 µM during in vitro maturation of aged oocytes	Decreased aberrant spindle organization, increases ATP production, increases the development of bovine oocytes and reduces apoptotic rat; downregulates BAX and CASP3 and increases BCL2	[99]
Rabbit	Morula	10^−3^ M prior in vitro culture, prior vitrification	Promotes blastocyst rate, increases SOD activity and decreases LPO and NO levels	[100]
Mouse	Preantral follicles	10 pM after vitrification, during culture	Increases diameter of follicles and their survival	[101]
Bovine	Embryos produced by SCNT	10^−11^ to 10^−2^ M during in vitro culture	Increases total cell number, ICM and the development of bovine SCNT embryos; suppresses the expression of *p53* and *Bax*, and upregulates *SOD1*, *Gpx4*, *BCL2L1* and *SOX2*	[102]
Porcine	Oocyte	10^−7^ M during in vitro maturation under heat stress	Improves polar body and blastocyst rate impaired by heat stress.; preserves normal levels of steroid hormone, reduces ROS, enhances GSH production and inhibits apoptosis	[103]
Porcine	Oocyte and embryos	25 ng/mL during in vitro maturation and culture	Increases blastocyst rate and decrease apoptotic nuclei in embryos	[104]
Bovine	Sperm	1000 nM	Increases higher wobbler coefficient, decreases sperm with intact acrosome and viable spermatozoa with ROS	[105]
Rabbit	Embryos	10^−9^ to 10^−3^ M during in vitro culture	Increases in vitro development and improves hatching rate	[106]
Bovine	Zygotes	10^−7^ M during in vitro culture	Promotes the cleavage and blastocyst rate, accelerates the development of in vitro embryos and improves the quality of blastocysts	[107]
Bovine	Zygotes	10^−7^ M for 2 days at the beginning of in vitro culture	Increases the blastocysts and hatched blastocyst rate	[108]
Bovine	Zygotes	10^−9^ M for after 2 days of pre-culture and for the remaining 6 days of culture	Increases the blastocysts and hatched blastocysts rate	[108]
Bovine	GV oocytes	10^−9^ or 10^−7^ M during in vitro maturation	Improves embryo development and the total cell number after in vitro fertilization; upregulates genes associated during in vitro maturation: *GDF9*, *MARF1* and *DNMT1α*	[109]
Mouse	2-cell embryos	10 µM during in vitro culture	Improves quality and developmental rate of embryos; can prevent cell death	[110]
Rat	Sperm	10 mg/kg weekly for 8 weeks	Improves sperm motility	[111]
Mouse	Embryos	10^−12^ M during in vitro culture of embryos produced by SCNT	Increases embryo development	[112]
Ovine	Blastocysts	10^−9^ M during thawing after cryopreservation	Improves embryo development after post warming culture	[113]
Deer	Animal	Subcutaneous implantation of 40 mg	Elevates serum FSH and LH levels, increases number of corpora luteal and the number of embryos	[114]
Sheep	Animal	Subcutaneous implantation of 40 or 80 mg	Increases corpus lutea, the number of recovered embryos, pregnancy and birth rates, and the number of lambs born per embryo	[115]
Porcine	Donor cell and embryos	10^−10^ M in the medium for donor cell and 10^−9^ M during in vitro culture of embryos produced by SCNT	Increases proliferation of fetal fibroblasts and the blastocysts rate,; reduces the apoptotic nuclei.; upregulates BCL2L1 and downregulates *BAX* and *p53*	[116]
Mouse	Oocytes	10 to 100 nM during in vitro maturation	Increases expansion, maturation, fertilization and blastocyst rate in a dose dependent manner	[117]
Bovine	Oocytes	10^−12^ to 10^−3^ M during in vitro maturation under heat stress	Increases blastocyst rate of embryos submitted to heat stress	[118]
Murine	Pronuclear embryos	10^−7^ M during in vitro culture	Promotes embryo development, blastocyst rate, hatching rate and blastocyst cell number; upregulates *SOD* and *BCL2* and downregulates *CAS3* and *p53*	[119]
